# Disparities and Menthol Marketing: Additional Evidence in Support of Point of Sale Policies

**DOI:** 10.3390/ijerph10104571

**Published:** 2013-09-25

**Authors:** Sarah Moreland-Russell, Jenine Harris, Doneisha Snider, Heidi Walsh, Julianne Cyr, Joaquin Barnoya

**Affiliations:** 1 George Warren Brown School of Social Work, Washington University in St. Louis, St. Louis, MO 63112, USA; E-Mails: jharris@brownschool.wustl.edu (J.H.); dsnider@gwbmail.wustl.edu (D.S.); hmonk@gwbmail.wustl.edu (H.W.); julianne.cyr.mph@gmail.com (J.C.); 2 Department of Surgery, Division of Public Health Sciences, Washington University in St. Louis, St. Louis, MO 63112, USA; E-Mail: barnoyaj@wudosis.wustl.edu

**Keywords:** point of sale, advertising, menthol, policy, disparities

## Abstract

This study examined factors associated with point-of-sale tobacco marketing in St. Louis, an urban city in the United States. Using spatial analysis, descriptive statistics, and multilevel modeling, we examined point-of-sale data and the proportion of mentholated cigarette and total cigarette marketing from 342 individual tobacco retail stores within St. Louis census tracts characterized by the percent of black adults and children. Menthol and total tobacco product marketing was highest in areas with the highest percentages of black residents. When examining menthol marketing to children, we did not find as strong of a relationship, however results of multilevel modeling indicate that as the proportion of black children in a census tract increased, the proportion of menthol marketing near candy also increased. These results indicate the need for communities globally to counter this targeted marketing by taking policy action specifically through the enactment of marketing restrictions provided by the 2009 Family Smoking Prevention and Tobacco Control Act and the Framework Convention of Tobacco Control.

## 1. Introduction

In the United States, the retail setting is a major venue where tobacco products are advertised and promoted. The tobacco industry spends billions of dollars annually, using the retail environment to maximize the availability and visibility of tobacco products, promote brand image and identity, implement price promotions that undercut the impact of tax increases, and, more generally, to normalize tobacco products and increase their use [1,2,3,4]. The industry also strategically focuses efforts within minority communities, using population-specific branding, marketing, and strategic placement. In 1998 the Master Settlement Agreement (MSA) banned most traditional advertising mechanisms (e.g., billboards, transit advertising, and sponsored events) for tobacco products. The retail environment, typically referred to as the point of sale (POS), became the main channel for the tobacco industry to advertise, promote, and market its products. By using strategies such as price discounting, indoor and outdoor advertising, and selling new products with alluring flavors, the industry is able to promote its products to a captive audience, normalizing tobacco use and prompting impulse purchases [5]. Black communities in particular have been subject to intense advertising and promotional efforts at the POS [6,7,8,9]. Tobacco outlet density is significantly higher in census tracts with greater black populations and lower household incomes [10,11,12]. Mentholated cigarettes, in particular, are marketed heavily to black communities [13,14], raising concerns for potential increased negative health consequences [15]. Menthol is derived from peppermint to produce a minty flavor and cooling sensation masking the tobacco taste and throat irritation associated with smoking [15,16]. Menthol cigarettes were quickly adopted by blacks (especially black youth and children) after the tobacco industry invested in aggressive marketing campaigns targeted at black communities [17]. Campaigns included advertising in magazines read in the black community, often by black youth (e.g., *Ebony*, *Jet*, *Essence*) [8,18,19,20,21], on billboards, and at the POS in minority neighborhoods. Studies on billboard advertising found black neighborhoods were significantly more likely than other neighborhoods to have menthol advertisements [13,22]. Stationary forms of POS tobacco advertising are also more likely to occur in minority communities [14,22,23] and in stores preferred by youth [24,25]. Laws and colleagues found that 29% of POS advertisements in minority neighborhoods were for menthol products, compared to 10% in non-minority neighborhoods [14]. The strategic advertising particularly of mentholated products, to and in black communities translates into increased initiation and use among blacks. Research shows that POS advertising is associated with encouraging youth to try smoking, making them significantly more likely to become smokers [26,27,28,29]. Menthol cigarettes are smoked more often by blacks compared to whites (AOR = 10.92; 95% CI = 9.58–12.44) and by young adults compared to the elderly (AOR = 1.39; 95% CI = 1.12–1.73) [30]. Approximately 80% of black smokers use menthol cigarettes [31], with the vast majority of black youth smoking the three most heavily advertised mentholated cigarette brands: Newport, Kool, and Marlboro [32].

While research is inconclusive on adult initiation of tobacco use and POS advertising exposure, Wakefield *et al*. [33] found strong evidence from self-reports of smokers and recent quitters of the impact of tobacco marketing and displays on prompting impulse buying and urges to smoke, and undermining quit attempts. These effects were greater among those who noticed POS displays more often, among women, and among smokers from disadvantaged areas [33]. 

Policies restricting or eliminating POS advertising have become increasingly important and more readily adopted. Nineteen countries have enacted complete bans on tobacco advertising, promotion, and sponsorship [34]; others have implemented extensive advertising restrictions, especially since the Framework Convention on Tobacco Control [35]. Unfortunately, this strategy has not been widely implemented in the United States due, in part, to First Amendment commercial speech protections [36]. The U.S. 1965 Federal Cigarette Labeling and Advertising Act (FCLAA) restricted advertising and promotion of cigarettes (but not other tobacco products) based on smoking and health concerns but preempted, or did not allow states and municipalities from enacting advertising restrictions stronger than those at the federal level [36]. This preemption was partially rescinded in 2009 when the Food and Drug Administration (FDA) was given the authority to regulate the manufacturing and marketing of tobacco products as part of the Family Smoking Prevention and Tobacco Control Act (FSPTCA). The partial elimination of FCLAA’s preemptions provides new opportunities for state and local governments to implement policies that reduce POS advertising and tobacco use [37]. To show the importance of widespread adoption of POS policies throughout the United States, it is important to understand the current retail environment. The purpose of this research is to examine the association between neighborhood-level racial characteristics and POS tobacco marketing. Specifically, this paper focuses on three hypotheses:

(1) Store characteristics (specifically store size and type) and neighborhood composition (specifically, total number of black residents and children) will be associated with increased POS tobacco marketing.
(2) There will be a positive relationship between the percentage of the community that is black and the proportion of tobacco marketing in stores that is menthol.
(3) Stores in neighborhoods with a higher percentage of black children will have a higher proportion of marketing near candy.

While several studies in the past have exposed the industry’s strategic advertising to and in black communities, there have been few recent reports (most studies were published in the early to mid-2000s) reporting on the current landscape of the retail environment. In addition, few studies have examined menthol-specific advertising and more specifically, menthol advertising near candy. This information is also extremely important as the FDA considers a ban on mentholated products. As part of the 2009 Tobacco Control Act, the FDA’s Tobacco Products Scientific Advisory Committee (TPSAC) was appointed to examine the impact of mentholated cigarettes on public health, particularly for youth, African Americans, Hispanics, and other racial and ethnic minorities. TPSAC recommended that the FDA ban sales of menthol cigarettes [38]. Despite this, the FDA has yet to take regulatory action [39]. The recommendation is still being considered by the FDA and, if any regulatory action is taken, public notice and a public comment period would be required [39].

## 2. Experimental Section

We used three strategies to examine the relationship between menthol marketing and neighborhood composition: (1) GIS spatial analysis, (2) descriptive statistics, and (3) multilevel modeling.

### 2.1. Data Collection and Management

We conducted secondary data analysis using POS marketing data from a random sample of 342 of the 1,229 geomatched tobacco retailers located throughout St. Louis City and St. Louis County, Missouri. St. Louis City and County are separate political and economic entities. The sample was defined based on an original list of 1,234 retailers, obtained from the Missouri Department of Mental Health’s Division of Drug and Alcohol Abuse. The list was composed of Missouri retailers that were operational in 2009 and was geocoded using USA Geocoding Service. There was an initial automatic match rate of 89% (1,106/1,234). Unmatched addresses were individually researched and manually geocoded. All but five retailers were eventually matched, resulting in a final match rate of over 99% (1,229/1,234). 

The POS assessment was conducted from April through June 2009 by two trained research staff from the Center for Public Health Systems Science at Washington University in St. Louis. A previously validated tool [40] was used to assess store-level characteristics, including number of cash registers (proxy for store size), store type (e.g., convenience store, pharmacy, *etc.*), and interior and exterior marketing and branded functional items (e.g., items such as floor mats with tobacco advertisements). Furthermore, cigarette advertising and branded functional items, location (e.g., within 6 inches of candy, near the counter), count, brand, and flavor (e.g., menthol, no flavor) were collected. Each staff member collected information independently and then compared observations immediately after the assessment. Reliability was not assessed due to the immediacy of review, however in cases of incongruity in data collection, the staff went back into the store to confirm results.

### 2.2. Analyses

To answer our research questions, we examined four main outcome variables: (1) total tobacco marketing; (2) proportion of menthol marketing; (3) proportion of tobacco marketing near candy; and (4) proportion of menthol marketing near candy. Total tobacco marketing is the total number of cigarette advertisements and branded functional items appearing in the interior and exterior of each store. Proportion of menthol marketing is total menthol cigarette advertisements and branded functional items divided by total tobacco marketing. Proportion of menthol marketing near candy is the sum of menthol marketing near candy divided by the total menthol marketing. Proportion of tobacco marketing near candy is the sum of cigarette advertisements and branded functional items within 6 inches of candy divided by total tobacco marketing. Proportions were used in the place of counts to control for store size and overall amount of marketing in a store.

#### 2.2.1. GIS Spatial & Descriptive Analyses

We collected the total number of black children and the total number of black residents in each census tract from the 2010 U.S. Census [41]. Table 1 displays the general population descriptives of St. Louis, Missouri. To determine the proportion of black children in each census tract, we divided the total number of black children by the total population of adults and children of all races within each census tract. GIS spatial and descriptive analyses were performed using ArcMap. The retailer location data (street addresses and zip codes) were geocoded using USA Geocoding Service to obtain longitude and latitudes (*x*, *y* coordinates) for each retailer; these locations were overlaid to the 2010 census tract shapefile to determine in which census tract the retailers were located. The exact location of retailers in each census tract was determined by GIS using the “select by location” function. This allowed us to attach census tract characteristics of the area in which the retailer was located to the retailer for our descriptive analysis. For the analysis overall, data were categorized using natural breaks.

**Table 1:** General population characteristics of the sampled area.

	St. Louis City	St. Louis County	Total Area
Total population (n)	227,248	524,526	751,774
Census tracts (n)	71	102	173
Black residents (%)	54.58	26.74	35.16
Black children (%)	15.6	7.82	10.17

#### 2.2.2. Multilevel Model Development & Analyses

We used multilevel modeling to examine the relationship between neighborhood composition at the census tract level (level 2) and tobacco marketing in stores within each census tract (level 1). There were 173 total census tracts, for an average of 1.98 stores per tract and a range of one to seven stores per tract. In order to determine whether a multilevel model was useful in explaining the POS marketing patterns, we calculated the proportion of variance in each outcome accounted for by characteristics of the census tract (level 2) [42]. The proportion of the variation accounted for at the census tract level was 9% (ρ = 0.09) for total tobacco marketing, 36% (ρ = 0.36) for the proportion of marketing that was menthol, 50% (ρ = 0.50) for the proportion of marketing near candy, and 25% (ρ = 0.25) for the proportion of menthol marketing near candy. This represents a substantial amount of the variation attributable to differences at the census tract level. Models were developed in two steps. Because the outcome variables were highly skewed (see Table 2), they were transformed to standardized scores before modeling. A null model, or model with no predictors, was developed first and then independent variables were added. Store size and type may influence the amount of tobacco marketing [40], however, in this case store size and type were strongly and significantly associated (F(6) = 167.1; R^2^ = 0.75), so we selected store size to account for these characteristics in the models. Model fit was assessed using the Akaike Information Criterion (AIC) and the Bayesian Information Criterion (BIC). AIC and BIC are measures of model fit accounting for the number of independent variables in the model; higher AIC and BIC indicate reduced model fit. Because they account for the number of independent variables, both AIC and BIC can be used to compare fit across models of different sizes [42]. R version 2.11.1 was used for all analyses. A total of 342 stores were sampled; Table 2 displays the general characteristics of the retailer sample.

**Table 2:** Tobacco marketing characteristics in St. Louis tobacco retailers sampled.

	St. Louis City	St. Louis County	Total area
**Retailers (n)**	138	204	342
**Store type (n)**			
*Supermarket*	9	26	35
*Small market*	60	31	91
*Convenience (gas)*	5	7	12
*Convenience (no gas)*	37	85	122
*Drug store*	11	29	40
*Liquor store*	5	15	20
*Other store*	11	11	22
**Store size (mean, SD)**	2.27 (2.51)	3.63 (4.42)	3.09 (3.83)
**Total tobacco marketing (mean, SD)**	15.25 (10.56)	11.55 (9.53)	13.04 (10.11)
Menthol marketing(mean, SD) *	0.41 (0.24)	0.29 (0.24)	0.34 (0.25)
Tobacco marketing near candy (mean, SD) *	0.02 (0.05)	0.01 (0.07)	0.02 (.07)
Menthol marketing near candy (mean, SD) *	0.04 (0.16)	0.02 (0.12)	0.03 (.14)

* Proportion.

## 3. Results

### 3.1. Total Menthol Marketing in Areas with Higher Percentage of Black Residents

**Figure 1:**
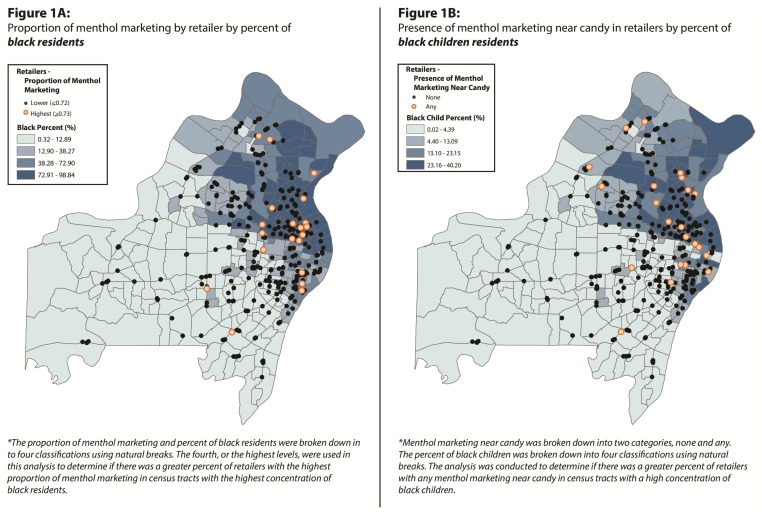
GIS Maps.

A proximity analysis in GIS identified a relationship between the number of black residents in a census tract and menthol marketing at the POS. Of the stores sampled, 81.87% of the retailers (n = 280) had some proportion of menthol marketing. Of those stores with any menthol marketing, 7.14% (n = 20) were classified as having the highest proportion (≥0.73 ads) of menthol marketing. Half (n = 10) of the retailers with the highest proportion of menthol marketing were located in census tracts that had the greatest percent of black residents (≥72.91%). In census tracts with the lowest percent of black residents (≤12.89%) only 5% of the retailers with the highest proportion of menthol marketing were present (Figure 1(A)). This analysis suggests that in areas with higher concentrations of black residents, there are more retailers with the highest proportion of menthol ads compared to areas with fewer black residents. Multilevel models also identified significant positive associations between the proportion of black residents (Table 3), marketing of all tobacco products, and proportion of menthol-specific marketing at stores by census tracts. That is, controlling for the size of the store, as the percent of black residents in a census tract increases, so does the total tobacco marketing in local stores and the proportion of that marketing that is menthol.

In addition, store size was not a significant predictor of total tobacco marketing. The AIC and BIC decreased over the null models, indicating the models with predictors were a better fit for the data than the null model.

**Table 3:** The association between tobacco marketing and menthol marketing at the point of sale and the concentration of black residents in census blocks.

	Model 1: Total tobacco marketing		Model 2: Proportion menthol marketing	
	b (s.e.)	*p*	b (s.e.)	*p*
Constant	1.67 (0.18)	<0.01	−2.67 (0.22)	<0.01
% black residents	0.01 (0.003)	<0.01	0.02 (0.004)	<0.01
Store size	−0.03 (0.03)	0.28	−0.03 (0.03)	0.31
Model fit	AIC = 961	BIC = 980	AIC = 907	BIC =926
Null model fit	AIC_null_ = 969	BIC_null_ = 980	AIC_null_ = 939	BIC_null_ = 950

### 3.2. Menthol Marketing near Candy and Proportion of Black Children

When examining the relationship between menthol marketing near candy at the POS and black children, we found that there was not a strong relationship between the proportion of menthol marketing near candy and the proportion of black children. That is, the percent of retailers with the highest proportion of menthol marketing near candy was the same (25%) in areas with the highest percent of black children (range = 23.16%–40.20%) and in areas with the lowest percent of black children (≤4.39%).

Only 12.87% of the retailers (n = 44) had some proportion of tobacco marketing near candy, with 23 having any proportion of marketing near candy that was menthol (Figure 1(B)). We examined whether the 23 retailers with any menthol marketing near candy were primarily located in areas with a greater percent of black children. To do this, we used the “select by location” function in GIS. All retailers with “any” menthol marketing near candy were selected if they were located in any census tract that had 23.16–40.20% of black children within the population. This was done for each category break of black children to determine which locations had retailers with “any” proportion of menthol ads near candy. A similar approach was taken for the menthol marketing in the black community. The proportions for this approach were broken down into 4 categories using natural breaks in order to understand the magnitude of menthol marketing. Only two categories are shown for better visualization (Figure 1(B)). In census tracts with the highest percentage of black children, 39.13% (n = 9) of the retailers with any proportion of menthol marketing near candy were present, while only 4.34% (n = 1) were located in census tracts with the lowest percentage of black children. Overall, retailers with any proportion of menthol marketing near candy are much more likely to be in census tracts with a higher percent of black children than in those with a lower percent of black children.

While multilevel modeling also showed that, controlling for store size, the proportion of tobacco marketing near candy was not significantly associated with the percent of black children in the neighborhood (Table 4—Model 3), as the proportion of black children in a census tract increased, the proportion of menthol marketing near candy also increased (*p* < 0.01) (Table 4—Model 4). Increases in AIC and BIC indicated a worse model fit compared to a null model with no predictors.

**Table 4:** The association between tobacco marketing at the point of sale and the proportion of black children in census tracts.

	Model 3: Proportion marketing near candy		Model 4: Proportion menthol marketing near candy	
	b (s.e.)	*p*	b (s.e.)	*p*
Constant	−6.28 (0.16)	<0.05	−6.68 (0.15)	<0.01
% black children	0.01 (0.008)	0.13	0.02(0.008)	<0.01
Store size	−0.05 (0.02)	<0.05	−0.02 (0.02)	0.35
Model fit	AIC = 978	BIC = 997	AIC = 979	BIC = 998
Null model fit	AIC_null_ = 962	BIC_null_ = 973	AIC_null_ = 965	BIC_null_ = 976

## 4. Discussion

The objective of this study was to assess neighborhood and retail store characteristics associated with POS marketing. Specifically, we examined whether retail store characteristics (size, type, and location) and percentage of blacks in the community was associated with tobacco marketing. Consistent with other studies, we found marketing of all tobacco products and menthol-specific marketing to be greater in areas with a higher proportion of blacks [6,7,8,9,13,14]. While other studies have shown an association with store size and the number of advertisements, these were not significant predictors of marketing in areas with higher percentages of black residents in our models. In addition, we examined whether percentage of black children in the community explained total tobacco marketing and proportion of menthol marketing near candy. Examination using spatial analysis to determine the distribution of stores with any menthol marketing near candy showed that a greater percent of retailers with menthol marketing near candy were located in census tracts with the highest percent of black children. Further, while the multilevel models were not conclusive given poor fit, they suggested a potential positive relationship between the proportion of menthol marketing near candy and the percent of black children in a census tract. Overall, these findings suggest that menthol is being advertised more heavily to black children compared to other children. To our knowledge, this is the first study to examine the patterns of menthol-specific marketing near candy specifically in black neighborhoods. Given our results, and because a vast majority of black youth smoke heavily marketed brands, this topic warrants additional study.

### Limitations

There are several limitations to this study. First, this study used a cross-sectional design to examine cigarette marketing at the POS in one U.S. metropolitan region. Even though our sample is not representative of the whole state of Missouri, it is likely to be generalizable in areas of the state with higher percentages of black residents as there are no differences in tobacco control marketing restrictions between the St. Louis area and the rest of the state. Additionally, natural breaks were used to show grouping that actually occurs rather than grouping that occurs within assigned limits. While this was useful in examining the data for this study it limits the generalizability of our results. It is important to have empirical research which covers marketing of all tobacco products (especially given the new popularity of smokeless tobacco products) in different parts of the United States (especially differences in rural and urban communities) and different countries. Furthermore, given the new provisions of the FSPTCA, tobacco marketing is likely to change and longitudinal research that examines which marketing restriction policies are most effective in benefiting the most adversely affected populations is clearly needed. In addition, the number of stores marketing near candy was small, which may have led to a lack of precision in multilevel models for these outcomes. Finally, limitations exist when geocoding tobacco retailer data. Although a list of tobacco retailers was provided to a professional geocoding service and a 99% match rate was attained, some of the retailers may not have been geocoded due to incomplete addresses, variations in street names, and variations in business names [43]. It is unknown how this limitation affects our results, however our sample size should counter some of these effects.

## 5. Conclusions

A convincing body of evidence demonstrates that tobacco-related health disparities are perpetuated by the large amount of marketing in minority areas. According to the WHO’s MPOWER report for reducing tobacco use and the National Cancer Institute’s 2008 monograph, The Role of the Media in Promoting and Reducing Tobacco Use, adopting and enforcing comprehensive bans on tobacco advertising and promotion can result in a considerable reduction in tobacco consumption across all populations. Our data confirm the existence of an extensive amount of marketing at the POS, especially in areas with larger black populations. With the recent adoption of the FSPTCA, American states and local communities have the opportunity to implement and enforce POS policies that restrict or eliminate tobacco advertising and promotion. Such comprehensive policy adoption is imperative not only in reducing tobacco consumption, but also in the ongoing effort to eliminate tobacco-related population disparities. Our research and previous publications support a ban on the advertisement, promotion, and/or sale of mentholated products. Such a ban may be the best method for reaching U.S. populations most adversely affected by tobacco.
